# Lipofilling effects after breast cancer surgery in post-radiation patients: an analysis of results and algorithm proposal

**DOI:** 10.1007/s00238-017-1311-1

**Published:** 2017-05-29

**Authors:** Manuel Debald, Thomas Pech, Christina Kaiser, Mignon-Denise Keyver-Paik, Gisela Walgenbach-Bruenagel, Joerg C. Kalff, Walther Kuhn, Klaus J. Walgenbach

**Affiliations:** 1 Department of Obstetrics and Gynecology, Centre for Integrated Oncology, University of Bonn, Bonn, Germany; 2 Division of Plastic and Aesthetic Surgery, University of Bonn, Sigmund-Freud-Str. 25, 53127 Bonn, Germany; 3 Department of Surgery, Centre for Integrated Oncology, University of Bonn, Bonn, Germany

**Keywords:** Lipofilling, Fat transfer, Breast cancer, Breast reconstruction, Radiation, Regenerative medicine

## Abstract

**Background:**

Lipofilling or autologous fat transfer is an established technique in plastic surgery. Herein, we describe the lipofilling effects after implant-based breast reconstruction in post-radiation patients and propose an algorithm for indication of lipofilling.

**Methods:**

Forty patients with a history of breast cancer were included in this retrospective analysis. Patients had undergone either breast conserving therapy or mastectomy. Twenty-six patients underwent additional radiation therapy. Patients were assessed using a post-radiation skin scoring classification.

**Results:**

In total, 68 lipofilling procedures were analyzed. Scar release, skin softening, improved quality of life, and improvement of post-radiation findings are results of lipofilling with a closed filtration system. In all patients with post-surgical radiation, an improvement of tissue quality was observed. Staging revealed that lipofilling improved mean post-radiation skin scores of 2.40 ± 0.89 to 1.21 ± 0.76 (*p* ≤ 0.000). There was no recurrence of breast cancer in our study patients.

**Conclusions:**

This study introduces an algorithm using lipofilling in reconstructive breast surgery and especially in post-radiation patients with low risks as well as very high acceptance in patients with various indications for this procedure. A regenerative aspect was also detectable in patients following radiation therapy and reconstruction. Lipofilling is a safe and effective procedure with a low incidence of minor complications. It is therefore a feasible method to resolve volume deficiencies and asymmetric results after oncologic breast surgery. Nevertheless, a prospective study has now been initiated focusing on the oncologic safety of lipofilling including ultrasound and radiological examinations to validate the findings of this initial study.

Level of Evidence: Level IV, therapeutic study.

## Introduction

Autologous fat transfer or “lipofilling,” the surgical transfer of fat removed by liposuction to areas of the body that need filling out, has become an established technique in aesthetic surgery. In recent years, it has also gained major interest following reconstructive surgery and especially after breast cancer surgery. During lipofilling, autologous fat is transferred from site A (e.g., abdomen, flanks, limbs) to site B in order to change the shape or gain a reconstruction, e.g., after breast cancer surgery. To date, most clinical studies on lipofilling of the breast have been concerned with the aesthetic outcome, procedure-associated complications, or have described the method itself. One main concern following lipofilling was the presence of radiological findings, such as calcifications, that could lead to unnecessary invasive diagnostics [1]. Recent findings by Petit et al. suggest that lipofilling is a feasible procedure that does not affect radiological follow-up in breast cancer patients [2]. Additionally, it is well known that all kinds of breast surgery, including reduction, augmentation, and flap reconstruction, may lead to fat necrosis and therefore to calcifications [3–5]. Chan et al. summarized that lipofilling to the breast is a promising tool for restoring the contour of the breast as well as increasing the breast volume with excellent aesthetic outcomes. Several techniques are available for harvesting fatty tissue, purifying fat grafts, and infiltrating the purified tissue. Yet, only limited data are available analyzing the oncologic outcome and the potential risk of reduced sensitivity of diagnostic methods of lipofilling after breast cancer surgery [2, 6, 7].

Here, we report our experiences and initial results with lipofilling using a closed filtration system (Puregraft®) for fat transfer in breast reconstruction following breast cancer surgery, and discuss the procedure, the clinical and aesthetic outcome, and the histological findings. A major focus was the analysis of the effect of lipofilling in irradiated tissue.

## Patients and methods

### Patients

Forty patients, who underwent lipofilling between April 2011 and March 2014 using the Puregraft® system (Cytori Therapeutics, Inc., San Diego, California) at the Division of Plastic and Aesthetic Surgery, University of Bonn Medical Center, were included in this initial study. All patients had a history of breast cancer, 26 patients underwent radiotherapy.

The following criteria were analyzed: patient data, especially regarding oncologic history; type of reconstruction; percentage of lipofilling per type of reconstruction; practicability of the technique; aspirated, purified, and transferred volume; complications; post-operative outcome including the take of the graft and estimated tissue loss (minimal, moderate, severe); and the aesthetic outcome. Scoring was performed to evaluate the regenerative aspects based on changes in irradiated tissue (Table 1).

**Table 1:** Grading of skin damages pre- and post-lipofilling in irradiated patients (adopted from Lopez et al. [8]

Definition	
Grade 0	Absence of differences between irradiated and nonirradiated skin
Grade 1	Minimal teleangiectasia, slight breast asymmetry, mild hyperpigmentation
Grade 2	Marked teleangiectasia, moderate hyperpigmentation, increased density and palpable firmness, mild edema
Grade 3	Partially confluent teleangiectasia, severe hyperpigmentation, severe edema, subcutaneous fibrosis with fixation
Grade 4	Totally confluent teleangiectasia, very marked density, retraction and fixation. Major aesthetic sequelae in treated breast

### Harvesting method and fat graft preparation method

**Fig. 1:**
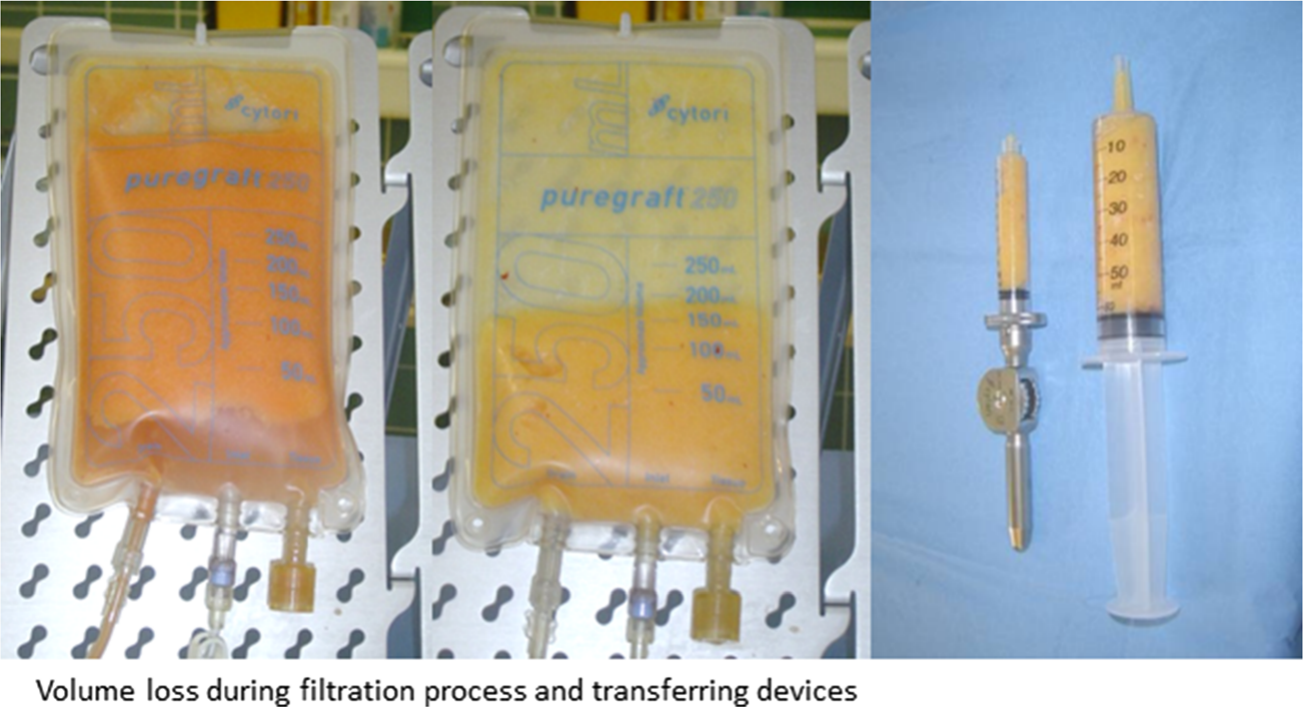
Filtration process

Patients received standard tumescent fluid infiltration of saline solution with lidocaine at 5 ml per liter, epinephrine at 1 ml per liter (1:1000) and 10 ml per liter bicarbonate (8.4%). Suction-assisted liposuction was performed with a Mercedes tip cannula (Byron) with 3 or 4 mm diameter. Lipoaspirate was processed with a closed filtrate system (Puregraft®) and washed with saline solution three to four times according to the manufacturer’s instructions. Processed material was retrieved from the system with a 60-ml syringe and transferred into 10-ml syringes into the Celbrush® device for lipofilling (Fig. 1). Next, the processed fatty tissue was injected using a microinjection multilayer technique to guarantee optimal distribution of the graft within the recipient site.

### Statistical analysis

Results are expressed as mean or median ± standard deviation. Analyses were performed using the Statistical Package for Social Sciences, version 22.0 (SPSS, Chicago, IL) and consisted of the nonparametric Wilcoxon test. *p* ≤ 0.05 was considered as significant.

## Results

### Oncologic history

**Fig. 2:**
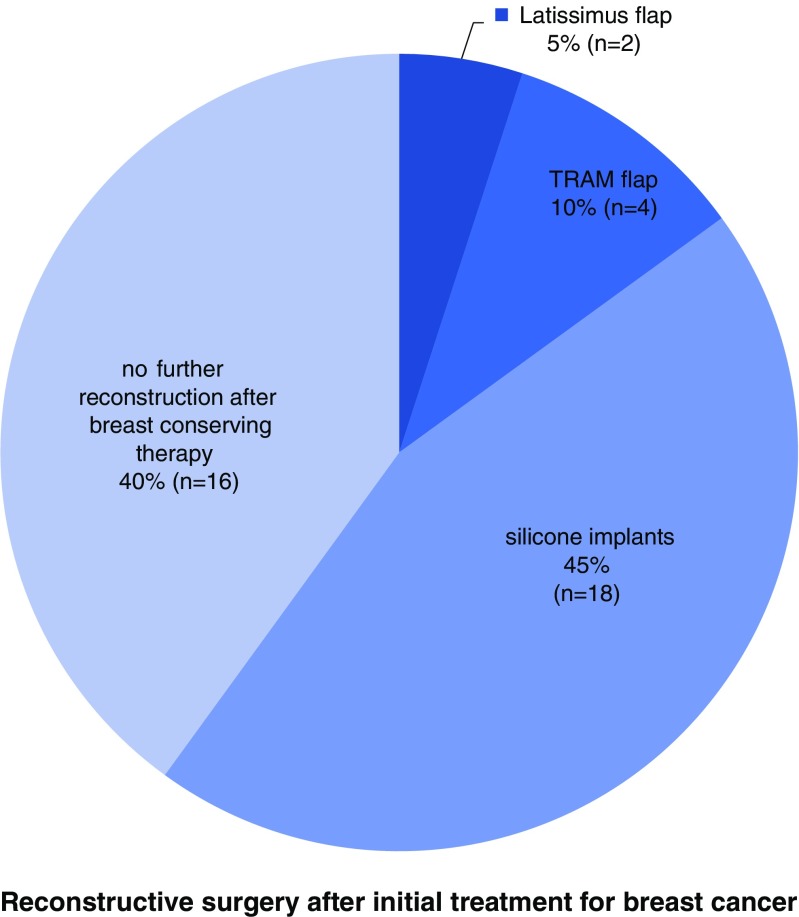
Type of aesthetic reconstruction after initial surgery for breast cancer (*n* = 40)

Tumor and patient characteristics at the time of initial breast cancer diagnosis are summarized in Table 2. In 23 patients (57.5%), a mastectomy was performed, whereas 17 patients (42.5%) underwent breast conserving therapy after breast cancer diagnosis. The types of reconstructive surgery after initial treatment for breast cancer are summarized in Fig. 2. In 26 patients (65.0%), radiotherapy was performed after initial surgery, after breast conserving therapy (BCT) or after mastectomy in cases with unfavorable factors indicating a high risk of recurrence. In 13 patients (32.5%), no radiotherapy was performed.

**Table 2:** Tumor and patient characteristics (*n* = 40)

Characteristics		No. (%)
Histology	Invasive lobular carcinoma	5 (12.5)
	Invasive ductal carcinoma	27 (67.5)
	Ductal carcinoma in situ	8 (20)
pT	Tis	8 (20)
	1	14 (35)
	2	11 (27.5)
	3	6 (15)
	4	1 (2.5)
pN	0	19 (47.5)
	1a	8 (20)
	2a	3 (7.5)
	3a	2 (5)
pM	0	39 (97.5)
	1	0 (2.5)

### Analysis of lipofilling

All patients (40/40) received lipofilling for contouring purposes after initial treatment for breast cancer. Lipofilling was performed on average after 71.6 months (range: 7–291.3; median: 43.4) after surgery for breast cancer and 25.9 months (range: 2.3–114.6; median: 20.3) after reconstructive surgery. The procedure was performed as in-patient procedure with a hospital stay of 1 to 2 days after lipofilling.

On average, the whole procedure lasted 65 min (range: 36–163; median: 69). Liposuction itself lasted on average 36 min (range: 21–52; median: 34), while fat transfer only lasted 18 min (range: 10–37; median: 17.5). In five patients (12.5%), an additional liposuction of the donor site or additional areas was performed for aesthetic purposes. Compared to the average operating time, these cases resulted in a prolonged operating time (54, 74, 75, 76, and 120 min), mainly due to prolonged liposuction times (32, 48, 50, 52, and 81 min). In one patient (2.5%), additional lipofilling was performed for aesthetic purposes to the hand (14 ml) and the face (two sites with 7 ml each). In this patient, lipoharvesting lasted 50 min, compared to 37 min for lipofilling.

### Liposuction sites

A liposuction of 818 ml total aspirate (range: 250–3730; median: 600) was performed on average. Donor sites varied with a focus on the abdomen and the flanks (Table 3). The sites of liposuction are summarized in Table 3. The lipoaspirate was processed by the Puregraft® system as mentioned above. After the filtration process, an average volume of 136 ml (range: 80–270 ml; median 125 ml) was available for lipofilling. This is equivalent to 16.6% of the initially harvested volume. On average, 123 ml of the filtrated lipoaspirate was transferred (range: 30–260; median: 120).

**Table 3:** Donor sites for liposuction (*n* = 40)

Site	No.
Flanks	4
Abdomen	19
Abdomen and flanks	11
Abdomen and flanks and upper limbs	1
Upper limbs	5

### Multiple procedures

In 25 patients (62.5%), a second lipofilling procedure was recommended due to a loss of volume at the site of fat transfer. All patients were already satisfied after the first procedure and recognized a significant improvement. Only 21 of 25 (84%) patients followed the professional recommendation for an additional procedure. In the end, only 21 of 40 patients (42.5%) underwent a further lipofilling procedure. The second procedure was performed on average 7.9 months (range: 2.7–26.9; median: 7) after the first lipofilling. In all of these cases, liposuction was performed at the abdomen and/or flanks. For the second procedure, a liposuction of 782 ml on average was performed (range: 400–2500; median: 700). On average, 149.6 ml were transferred (range: 60–270; median: 145). The average time for the whole second procedure was 68 min (range: 45–85; median 60), with 36 min (range: 26–51; median: 39) for the liposuction and 18.8 min (range: 10–33; median: 18.5) for the fat transfer. In three patients (7.5%), a third and in one patient (2.5%), a fourth procedure was indicated. This was also due to loss of volume. In total, 68 lipofilling procedures were performed. The duration of the filtration process had almost no negative influence on operating times, especially in patients receiving larger volumes. The filtration could be easily performed by the assisting personnel while lipofilling was already underway.

### Aesthetic outcome and follow-up

For 38 (95%) patients, follow-up data were available. Two patients (5%) were lost to follow-up. Patients were recommended to visit the clinic within 2–3 weeks after the procedure for wound control and clinical examination. On average, the first follow-up visit was 23.4 days (range: 6–100; median: 15) after lipofilling. At that time point, all patients as well as the surgeon were satisfied with the aesthetic results of the procedure. A mild form of hematoma in combination with swelling and redness, an omnipresent occurrence after liposuction (also in aesthetic liposuction), was observed in the patients we investigated. In one patient (2.5%), excessive hematoma and associated pain were observed at the site of liposuction. A further occurrence was a loss of volume at the site of fat transfer. As mentioned above, a second procedure was performed in 21 patients, while four patients underwent several procedures due to repetitive loss of volume at the site of fat transfer. Of note, apart from the improved aesthetic outcomes, patients who underwent radiotherapy reported a continuous softening of the breast after lipofilling was performed.

### Post-procedure histology

**Fig. 3:**
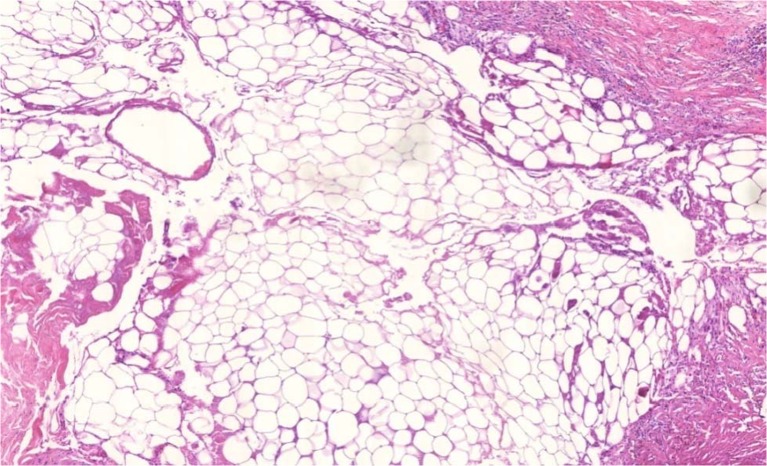
Histological specimen 10 months after lipofilling procedure

Representative specimens were taken 10 months after lipofilling during revision surgery for scar release. The histologic examination revealed the presence of viable univacular adipose tissue and only small areas of isolated fatty necrosis (Fig. 3). The transferred fat cells were embedded within the regular fibrous and adipose tissue, and blood vessels were sprouting into the newly formed adipose tissue. No histological evidence for recurrent disease or malignant tissue transformation was detected.

### Regenerative aspects

In all patients who had previously received radiation therapy in addition to the surgical oncological treatment (*n* = 26), we observed a significant improvement in softness of tissue and scars combined with an increase in comfort and quality of life. Patients described a release of hardening and rigidity of the treated breast as well as an increased mobility of the implant and the entire surrounding breast tissue.

**Fig. 4:**
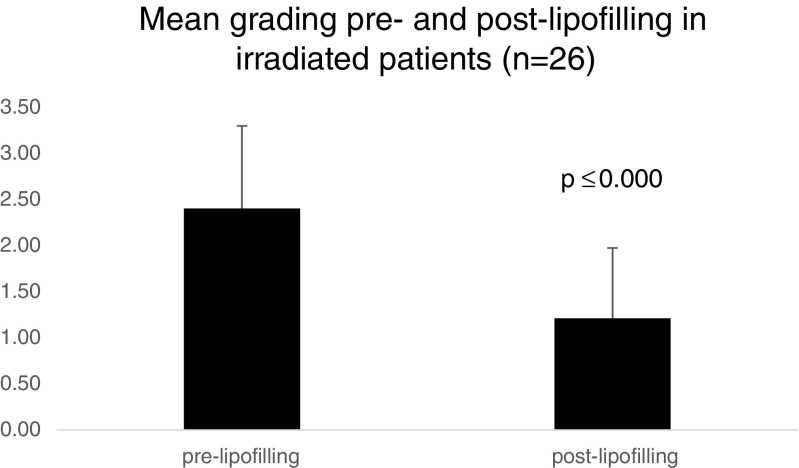
Grading of skin damages pre- and post-lipofilling in irradiated patients

In the staging for post-radiation skin and tissue damage, patients improved significantly (*p* ≤ 0.000) with ameliorated scores from 2.40 ± 0.89 before lipofilling to 1.21 ± 0.76 after lipofilling (Fig. 4).

## Discussion

Lipofilling in patients with reconstruction following breast cancer treatment has gained major interest. Based on our initial results using a closed filtration system, lipofilling is a patient-friendly technique providing new personalized reconstructive options with excellent aesthetic outcome and very low complication rates.

**Fig. 5:**
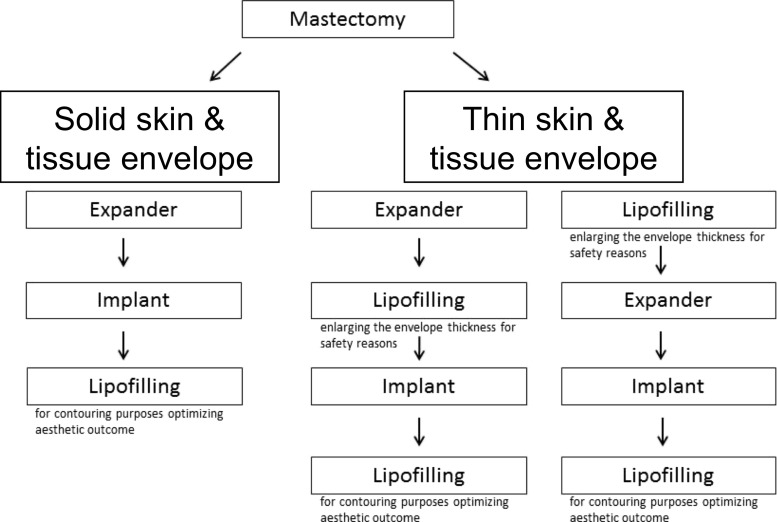
Algorithm for indication of lipofilling in expander/implant reconstruction

Lipofilling is a perfect tool to optimize aesthetic outcomes of different reconstructive plastic surgery approaches. In most cases, lipofilling will be performed after implants have been placed following possible skin extension using expanders (Fig. 5). Nevertheless, lipofilling can also be very helpful after autologous reconstruction.

The general indications for lipofilling are volume deficiencies and asymmetric results after implant or autologous tissue reconstruction (transverse rectus abdominis myocutaneous (TRAM), deep inferior epigastric perforators (DIEP), latissimus flap, etc.) to correct or improve the aesthetic outcome. New findings regarding improvement of post-radiation skin alterations or scar tissue itself with skin softening and scar release without resection resulted in a broadening of the indications for lipofilling. Additionally, general increase of tissue thickness can also be a valid indication for lipofilling. In this context, Sarfati et al. propose the use of lipofilling after radiotherapy to the breast in order to achieve better conditions for breast implant reconstruction [9, 10].

There are several aspects regarding post-radiation patients. In general, lipofilling may allow the reconstruction with the use of an expander followed by an implant as it can enhance the tissue thickness before the first step of breast reconstruction. In the present study, this situation was present in one case. It is a procedure that should be considered in patients with very thin and scary tissue after mastectomy. After lipofilling, post-radiation tissue becomes subjectively and objectively softer and, in addition to an improved aesthetic outcome, this improves the quality of life in the patient. The mean score improved significantly, while in particular, patients with high pre-lipofilling grading results benefitted the most. All in all, lipofilling in post-radiation tissue is a step towards a true regenerative aspect in reconstructive breast surgery. Patients who underwent pedicled TRAM or free DIEP skin flap reconstruction procedures also benefit from lipofilling. While the correction of asymmetries and contour deficiencies are major indications, in some cases with defects after partial necrosis, lipofilling appears to be the perfect method to optimize local problems, when volume deficiencies occur at sites which are not accessible for implants, or for other local corrections.

In patients with simple and small oncological resections (after breast conserving therapy), lipofilling may be the only approach to successful breast remodeling.

**Fig. 6:**
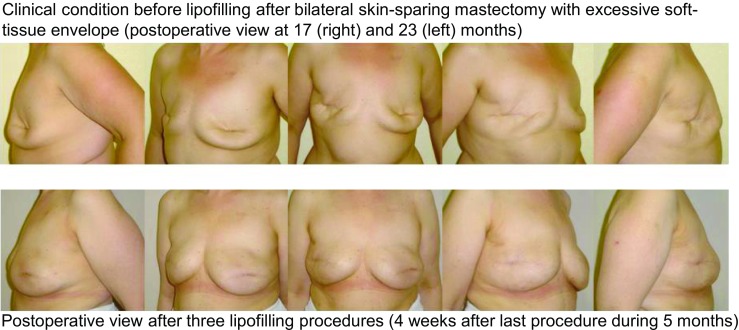
Clinical example of aesthetic outcome after lipofilling

In general, there is a demand for a more individualized reconstruction. Patients with small breast volume and thus possibly unfavorable options for implant reconstruction can also benefit from lipofilling without further reconstructive procedures. Even with higher volume differences, multiple lipofilling procedures can be applied easily in most of these patients and will be tolerated well because of low pain levels and short mean hospital stays (clinical example—Fig. 6). Panettiere et al. describe a case of large breast reconstruction only by lipofilling to the breast in nine sessions with excellent aesthetic outcome [11]. This approach is especially beneficial in patients with contraindications to flap use or a lack of common reconstruction options. Multiple donor sites for fat transfer are available, which allows multiple procedures also in patients with low BMI. However, the surgeon must carefully calculate the amount of the expected graft tissue. During the cleaning and washing process, approximately two thirds of the initially aspirated total volume will be lost. New filtration systems may change this rate in the future. Another aspect that requires consideration is the still unpredictable loss due to tissue necrosis after lipofilling. Obviously, in patients with low BMI and thin subcutaneous layers, liposuction for harvesting has to be extremely precise to avoid additional aesthetic problems at the harvest site.

Lipofilling can be performed in relative short and safe procedures. Here, we describe an average incision-to-suture time of 65 min. Based on the finding that the time for the liposuction (36 min) was twice as long as the fat transfer (18 min) and the fact that complications occur mainly at the harvesting site, lipobanking could be a promising tool to decrease procedure times and minimize complications. As the fat transfer should be performed in multiple small depots to achieve minimal diffusion distances, a stepwise augmentation would also be beneficial to obtain a reasonable amount of viable engraftment [12, 13]. Based on our experience, the average transfer of 123 ml per breast resulted in excellent aesthetic outcomes and patient satisfaction, while higher volumes (up to 260 ml in the present analysis) are also feasible. While every patient obviously requires an individual approach, we noted a learning curve regarding the suitable amount of injected tissue for each of the possible indications. Lipoharvesting can be performed at every part of the body without any deficits regarding the adipocyte quality or cell adhesion of the transplant [14]. Thus, the harvesting site can also be chosen regarding aesthetic aspects resulting in improved patient satisfaction. The surgeon should be experienced in liposuction since the aesthetic outcome on the donor site is also of clinical significance.

Using lipofilling, we were able to offer this method to patients after breast conserving therapy as well as mastectomy. In the abovementioned patients, no recurrence was observed after lipofilling. Moreover, no difficulties in radiologically differentiating scar or fat necrosis from normal breast tissue after lipofilling were noted. This is in agreement with previous studies, which did not reveal any problems in radiological breast examinations following fat transfer to the breast [2, 15]. In patients with extremely thin skin and fatty tissue layers, lipofilling can also be performed prior to reconstructive surgery to prevent skin defects or ulcerations, especially after radiotherapy. The 26 patients with previous radiotherapy also described skin softening and less paresthesia after lipofilling, which also enhances the functional outcome and minimizes the risk of local skin complications. In addition, softening of scar tissue was observed by most of the patients with or without previous radiotherapy, especially when scar tissue was present at multiple sites of the breast or after multiple previous procedures. In these cases, protective lipofilling increases the options for implant-based reconstruction and may alter conditions in patients who would otherwise not be candidates for this type of reconstruction [16]. Similar findings have been made by two recent studies with significant benefits due to lipofilling for patients after radiotherapy [17, 18].

While the underlying mechanisms are not yet fully understood, the observations described together with the clinical findings show the true regenerative aspects of lipofilling and autologous fat transfer. A recent animal study also confirmed these findings, such as positive effects on skin thickness, in a murine model [19]. Further studies must be carried out to evaluate whether the effect is due to the presence of adipose tissue-derived stem cells or to the general release of mediators, such as growth factors, within the healing process.

This is in line with findings of Schultz et al., who described an improved shape and consistency of the breast after lipotransfer to the breast [20]. Irradiated tissue or scar tissue must be released with a blunt cannula during microinjection. Complete scar release or scar excision immediately prior to the fat grafting with consecutive formation of a cavity is not possible since the injected fatty tissue would become necrotic. It will be necessary to perform several stage procedures due to contracture of oblique mastectomy scars with small advances in tissue quality. Nevertheless, the increase in tissue diameter can sometimes only be achieved stepwise since the transferred fat cells need healthy tissue with sufficient blood supply at the recipient site to survive the lipofilling procedure. In implant reconstruction, patients with extremely thin, irradiated skin, a very careful injection is indicated in order to avoid implant damage. Nevertheless, these patients will benefit especially from lipofilling.

Lipofilling considerably expands the horizon of implant-based breast reconstruction. It provides new opportunities in situations that had previously been considered unfavorable for implants, such as thin tissue layers and post-radiation conditions. Additionally, lipofilling after radiotherapy results in a significant improvement of skin quality and tissue regeneration. In our opinion, in combination with a well-considered clinical and radiological follow-up, lipofilling can be a safe and promising procedure after breast cancer surgery.
